# Characterization of selected organometallic compounds by electrospray ionization‐ and matrix‐assisted laser desorption/ionization‐mass spectrometry using different types of instruments: Possibilities and limitations

**DOI:** 10.1002/rcm.9281

**Published:** 2022-03-21

**Authors:** Sarah Fleissner, Ernst Pittenauer, Jan Pecak, Karl Kirchner

**Affiliations:** ^1^ Institute of Applied Synthetic Chemistry Vienna University of Technology Vienna Austria; ^2^ Institute of Chemical Technologies and Analytics Vienna University of Technology Vienna Austria

## Abstract

**Rationale:**

Organometallic compounds are becoming increasingly important in their industrial application as catalysts. Mass spectrometry is an essential tool for the structural confirmation of such organometallics. Because the analysis of this class of molecules can be challenging, the ionization behavior and structural confirmation of selected transition metal catalysts are described in this work.

**Methods:**

The transition metal catalysts investigated were analyzed using classical vacuum MALDI reflectron TOF‐MS as well as intermediate pressure matrix‐assisted laser desorption/ionization quadrupole time‐of‐flight mass spectrometry (MALDI QTOF‐MS). Obtained mass spectra were compared with electrospray ionization MS (ESI‐MS) already established for organometallic compounds, utilizing a QTOF mass spectrometer here. In addition, various sample preparations, including two selected MALDI matrices (*trans*‐2‐[3‐(4‐*tert*‐butylphenyl)‐2‐methyl‐2‐propenylidene]malononitrile and 2,2′:5′,2″‐terthiophene) with different solvent combinations for MALDI‐MS measurements, were investigated in detail with respect to their correct isotope distribution of the molecular ions observed.

**Results:**

All investigated organometallic compounds were successfully identified by vacuum and intermediate pressure MALDI‐MS. Accurate masses of ions related to molecular ion species (e.g., [M‐Cl]^+^, [M]^+^, and [M + Na]^+^) could be determined by MALDI QTOF‐MS measurements with a mass error of less than ±5 ppm for all compounds. Both investigated MALDI matrices performed equally on both instruments. The impact of the analyte/matrix solvent mixtures turned out to be crucial for a successful analysis of the investigated compounds. In contrast, ESI QTOF‐MS yielded masses of ions related to molecular ion species in favorable cases.

**Conclusions:**

The use of MALDI‐MS for the structural confirmation of organometallic compounds is still not widely used. Nevertheless, this work showed that this analytical technique does have some benefits. The analysis of neutral catalysts proves to be quite useful, concluding that this technique provides a complement and/or an alternative to ESI‐MS.

## 1 INTRODUCTION

The development of efficient and sustainable organometallic catalysts for chemical transformations of simple/small organic molecules is an important part of current research.^1^, ^2^ There is a wide selection of literature dealing with the structural elucidation and analysis of these coordination compounds.^3^, ^4^, ^5^ In the field of mass spectrometry (MS), electrospray ionization (ESI) MS, which is considered a soft ionization technique, has already been established in the analysis of this substance class.^3^, ^4^, ^6^, ^7^ Despite convincing advantages such as fast and easy sample preparation, the analysis of neutral organometallic complexes with ESI‐MS is still a challenging task.^8^ Over the past few decades, matrix‐assisted laser desorption/ionization in combination with reflectron time‐of‐flight mass spectrometry (MALDI TOF‐MS) became a well‐established analytical technique in the field of biochemistry and bioanalysis.^9^, ^10^ It is known for its ability to study particularly large complex molecules (>300 kDa).^11^ Research performed using MALDI‐MS in the area of organometallic compounds, which are typically rather small molecules, is still rare.^12^, ^13^, ^14^ Therefore, the analysis with MALDI‐MS has some advantages that could compensate for the disadvantages of ESI‐MS: neutral compounds can be detected more easily.^8^

The current work investigates the ionization behavior of selected neutral catalysts in combination with some structural characterization by classical vacuum MALDI TOF‐MS as well as intermediate pressure MALDI‐MS utilizing a hybrid quadrupole/reflectron TOF instrument (MALDI QTOF‐MS) with the ability of high‐resolution/accurate mass determination. In addition, different MALDI matrices and the influence of various solvent mixtures for the sample preparation of different organometallic compounds are compared. Furthermore, the obtained results are compared with data obtained with ESI‐MS utilizing a hybrid quadrupole/reflectron TOF instrument (ESI QTOF‐MS) with the ability of high‐resolution/accurate mass determination of these samples. The investigated coordination compounds were selected with a focus on first‐row transition metal (V, Cr, Mn, Fe, Co, and Ni) pincer complexes, which are already established in homogeneous catalysis.^15^, ^16^, ^17^

## 2 EXPERIMENTAL

### 2.1 Chemicals and materials

**FIGURE 1:**
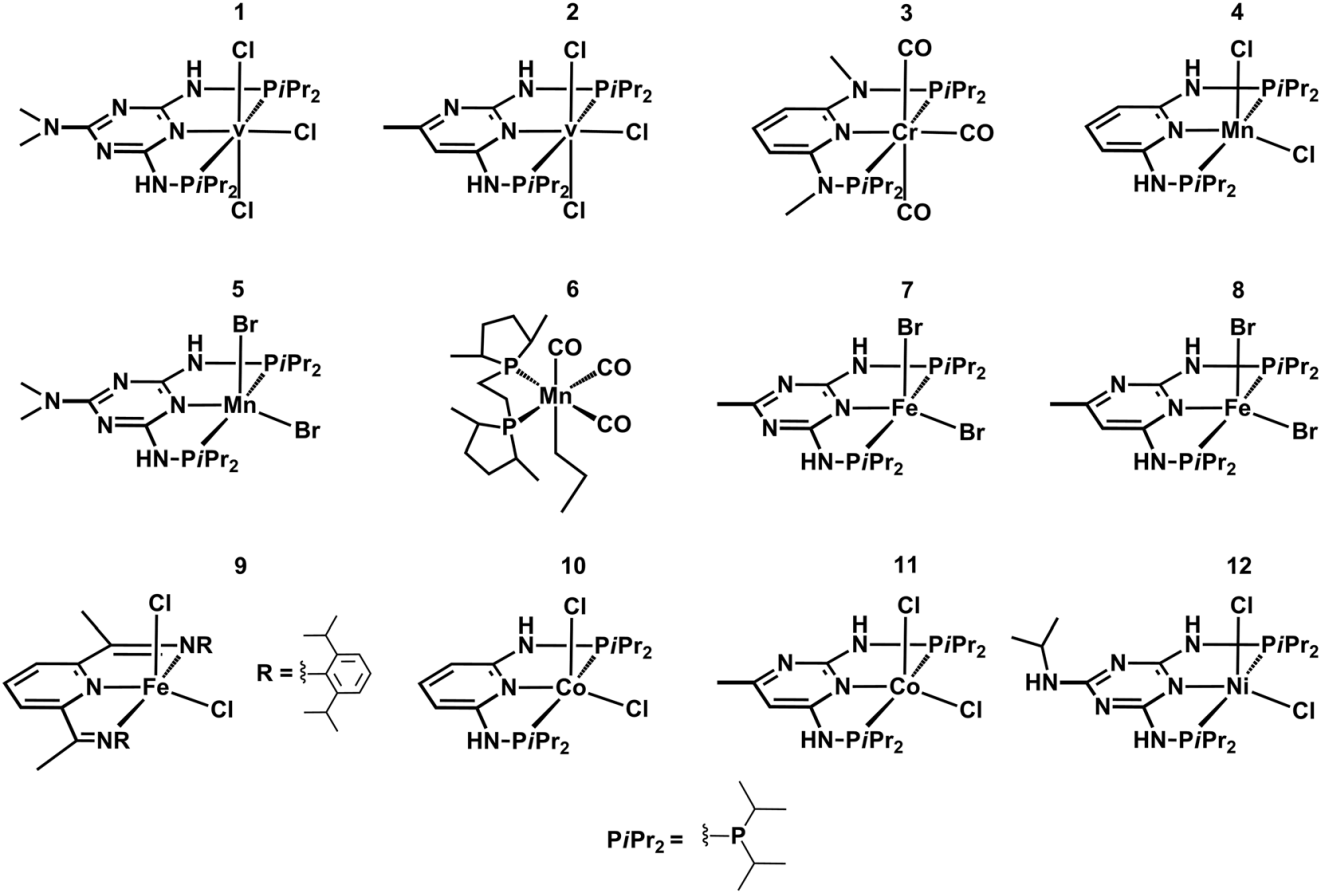
Chemical structures of the investigated analytes

All the analytes used in this work were synthesized and provided by our research group.^15^, ^16^, ^17^ These are bench‐stable compounds, where no special treatment under an inert gas atmosphere is required. The chemical structures of the organometallic catalysts selected for mass spectrometric investigation are shown in Figure 1, and the corresponding notations are presented in Table 1. Further information on the nomenclature of pincer complexes is available in the supporting information (Figure S1).

**TABLE 1:** Notation of the investigated samples

	Notation
1	[V(Triaz^NMe2^‐*i*Pr)Cl_3_]
2	[V(Pym^Me^‐*i*Pr)Cl_3_]
3	[Cr(PNP^NMe^‐*i*Pr)(CO)_3_]
4	[Mn(PNP^NH^‐*i*Pr)Cl_2_]
5	[Mn(Triaz^NMe2^‐*i*Pr)Br_2_]
6	[Mn(me‐bpe)(CO)_3_(C_3_H_7_)]
7	[Fe(Triaz^Me^‐*i*Pr)Br_2_]
8	[Fe(Pym^Me^‐*i*Pr)Br_2_]
9	[Fe(NNN‐PDI)Cl_2_]
10	[Co(PNP^NH^‐*i*Pr)Cl_2_]
11	[Co(Pym^Me^‐*i*Pr)Cl_2_]
12	[Ni(Triaz^NiPr^‐*i*Pr)Cl_2_]

The MALDI matrix *trans*‐2‐[3‐(4‐*tert*‐butylphenyl)‐2‐methyl‐2‐propenylidene]malononitrile (DCTB, ≥99.0%) was purchased from Merck (Darmstadt, Germany), and 2,2′:5′,2″‐terthiophene (TTP, ˃98%) was purchased from TCI Chemicals (Tokyo, Japan). The solvents acetonitrile (ACN), chloroform, and ethanol ACS Reag. Ph. Eur. were obtained from Merck; 1,2‐dichloropropane (>99.8%) from Fluka (St. Gallen, Switzerland); dichloromethane (>99.3%) from VWR (Radnor, PA, USA); methanol (≥99.9%) from Honeywell (Morristown, NJ, USA); and acetone (>99.5%) from Sigma‐Aldrich (St. Louis, MO. USA). The solid sodium chloride pro analysis was obtained from Merck and sodium bromide (>99.5%) from TCI Chemicals.

Red phosphorus, which was used for calibration in MALDI‐MS for both types of instruments, was obtained from Sigma‐Aldrich.^18^ ES tuning mix, used for calibration of the 6545 QTOF, was purchased from Agilent Technologies (Santa Clara, CA, USA).

### 2.2 Preparation of samples and solutions

The different solvent mixtures used for MALDI‐MS experiments are presented in Table 2. Both matrices, DCTB and TTP, were dissolved in the mentioned solvent mixtures resulting in a matrix concentration of 10 mg/mL. (Information on the chemical structure of the matrices used can be obtained from the supporting information [Figure S2].) The prepared matrix solution was then added to the solid analyte and mixed in a vortexer with an analyte concentration of 10 mg/mL. All final matrix/analyte solutions were doped with sodium chloride and sodium bromide (0.6 mg/mL), respectively. The type of salt to be added depends on the chemistry of the ligand(s) of the investigated sample. If a bromine atom is present as a ligand, sodium bromide was added, and for complexes with a chloride ligand or no halide ligand, sodium chloride was used. An aliquot (0.7 μL) of these sodium‐doped solutions was then applied onto the MALDI target by using the dried‐droplet method.^19^

**TABLE 2:** Used solvent mixtures for MALDI‐MS experiments

	Solvent 1	Solvent 2	Volume ratio (v/v)
1	Acetonitrile	Dichloromethane	1:1
2	Methanol	Chloroform	
3	Methanol	Dichloromethane	
4	Chloroform	1,2‐Dichloropropane	

For ESI‐MS sample preparations, solvent mixtures of ACN/dichloromethane, methanol/dichloromethane, and methanol/chloroform (v/v, 70:30) were prepared. All analytes were dissolved in these solvent mixtures resulting in a final analyte concentration of 0.1 mg/mL. A sodium salt was then added to the solution according to the selection criterion mentioned earlier.

### 2.3 Mass spectrometry

All MALDI‐MS experiments were performed on two different instruments, a Waters Synapt G2 (Waters, Manchester, UK) and a Bruker ultrafleXtreme (Bruker Daltonics, Bremen, Germany).

The Synapt G2 HDMS instrument was used in the intermediate pressure MALDI mode, where the ion source is operated under a pressure of ~2 × 10^−1^ mbar. The instrument is equipped with a 1 kHz Nd:YAG laser operating at a laser wavelength of 355 nm (frequency‐tripled), which has a Gaussian laser beam profile. It consists of a quadrupole, a so‐called Triwave section (for ion mobility measurements), and an orthogonal acceleration‐TOF mass analyzer. In this work the instrument was used in the single reflectron TOF mode. The measurements were performed in positive ion mode with a scan rate of 1 s per scan, a laser fluence of 320 arbitrary units (au, maximum 500 au), and a total acquisition time of 90 s for each run. Red phosphorus was used to calibrate the instrument and perform a lock mass correction after each sample run.^18^ Data acquisition and evaluation was performed using the software MassLynx v.4.1.

The ultrafleXtreme instrument is a high‐vacuum MALDI tandem TOF mass spectrometer, where ionization occurs under a pressure of ~2 × 10^−6^ mbar. The instrument is equipped with a 2 kHz smartbeam II laser system with a near‐rectangular beam profile. The laser is a frequency‐tripled Nd:YAG laser operating at a wavelength of 355 nm. The instrument can be set up for both linear and reflectron modes. All measurements were performed in positive ion reflectron mode with a frequency of 1 kHz, 1000 shots per final spectrum, and a laser fluence of 40%. Again, as a calibrant, red phosphorus was used. Spectra were processed using the flexAnalysis v3.4 software.

All ESI‐MS experiments were carried out on a 6545 QTOF mass spectrometer (Agilent Technologies) equipped with a standard electrospray ion source. It consists of a quadrupole, an rf‐only quadrupole collision cell, and a dual stage reflectron TOF mass analyzer. This work utilized the dual electrospray ionization source. All mass spectra were obtained in positive ion mode with an ion source temperature ranging from 220 to 240°C. For calibration, the ES tuning mix from Agilent (vide supra) was used. The obtained data were processed using the MassHunter Workstation Qualitative Analysis 10.0 software (Agilent). The sample was introduced to the system via direct infusion utilizing a syringe pump‐type model 100 (KD Scientific, Holliston, MA, USA).

All complexes included in this work were measured by HR‐MALDI QTOF‐MS as well by HR‐ESI QTOF‐MS with a mass error of less than ±5 ppm.

Simulations of resolution‐dependent isotope pattern distributions were carried out using the freeware IsoPro 3.0 (http://members.aol.com/MS; now sites.google.com).

## 3 RESULTS AND DISCUSSION

Analyzing coordination compounds by either MALDI or ESI‐MS, the identity of the elemental composition related to their chemical structure can be identified based on not only high‐resolution/accurate mass determination but also their specific isotope pattern. Many transition metals (chromium, iron, and nickel), but also ligands such as halogens (chlorine and bromine), have a specific isotope pattern whose presence in the mass spectrum is decisive for the identification of a particular substance. All analytes investigated and shown in Figure 1 constitute first‐row transition metal pincer/bidentate complexes, with bromine or chlorine as co‐ligands except for **3** and **6**.

**FIGURE 2:**
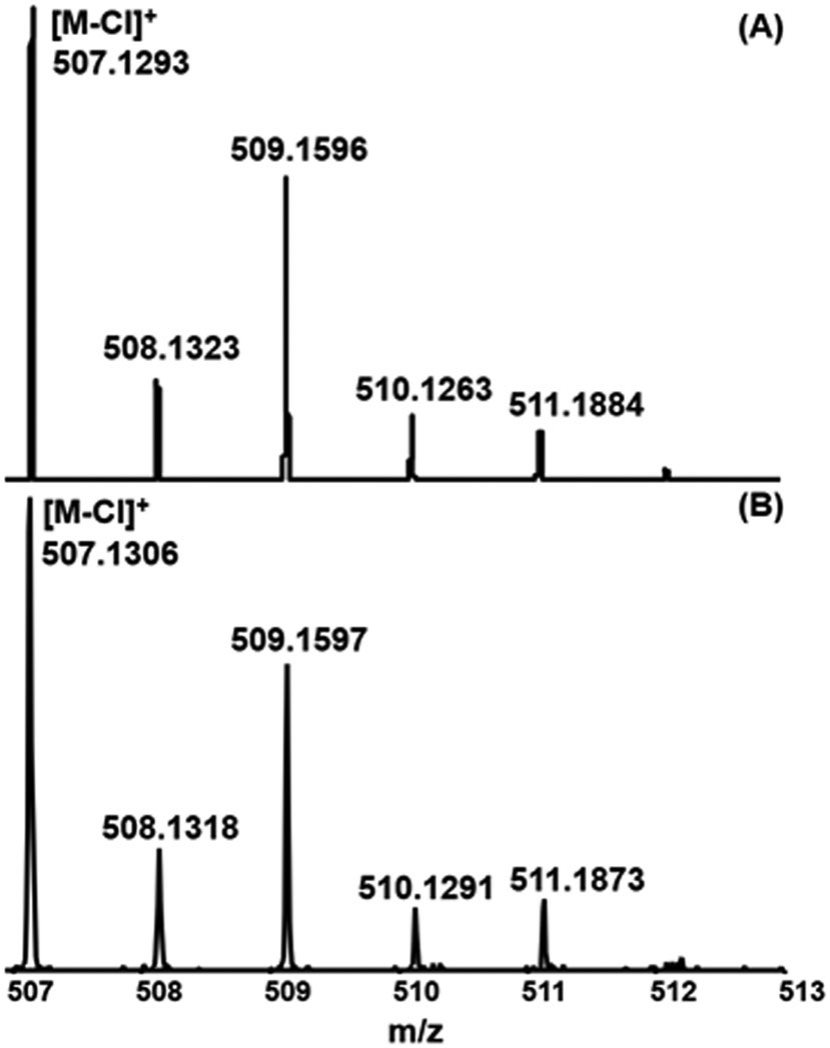
Comparison of the A, simulated and B, observed isotope pattern of **1** detected as dechlorinated molecule [M‐Cl]^+^ using HR‐MALDI QTOF‐MS

In the course of this work, isotope patterns of the identified molecular species were compared with the simulated ones. This is explained in detail here using **1** [V(Triaz^NMe2^‐*i*Pr)Cl_3_] as an example. Figure 2B represents the part of the spectrum containing the isotope pattern of the complex identified as dechlorinated molecule [M‐Cl]^+^, whereas Figure 2A shows the calculated isotope pattern for this species. A comparison of the two spectra (simulated and measured) shows that both the isotope patterns in combination with accurate mass determination of the monoisotopic species (Δ*m* = +2.5 ppm) agree very well with the calculation. In comparison, no molecular ions or ions related to the dechlorinated molecule [M‐Cl]^+^ could be assigned with ESI‐MS for this compound.

### 3.1 Performance differences between the Brønsted (Lewis) base matrices DCTB and TTP

**FIGURE 3:**
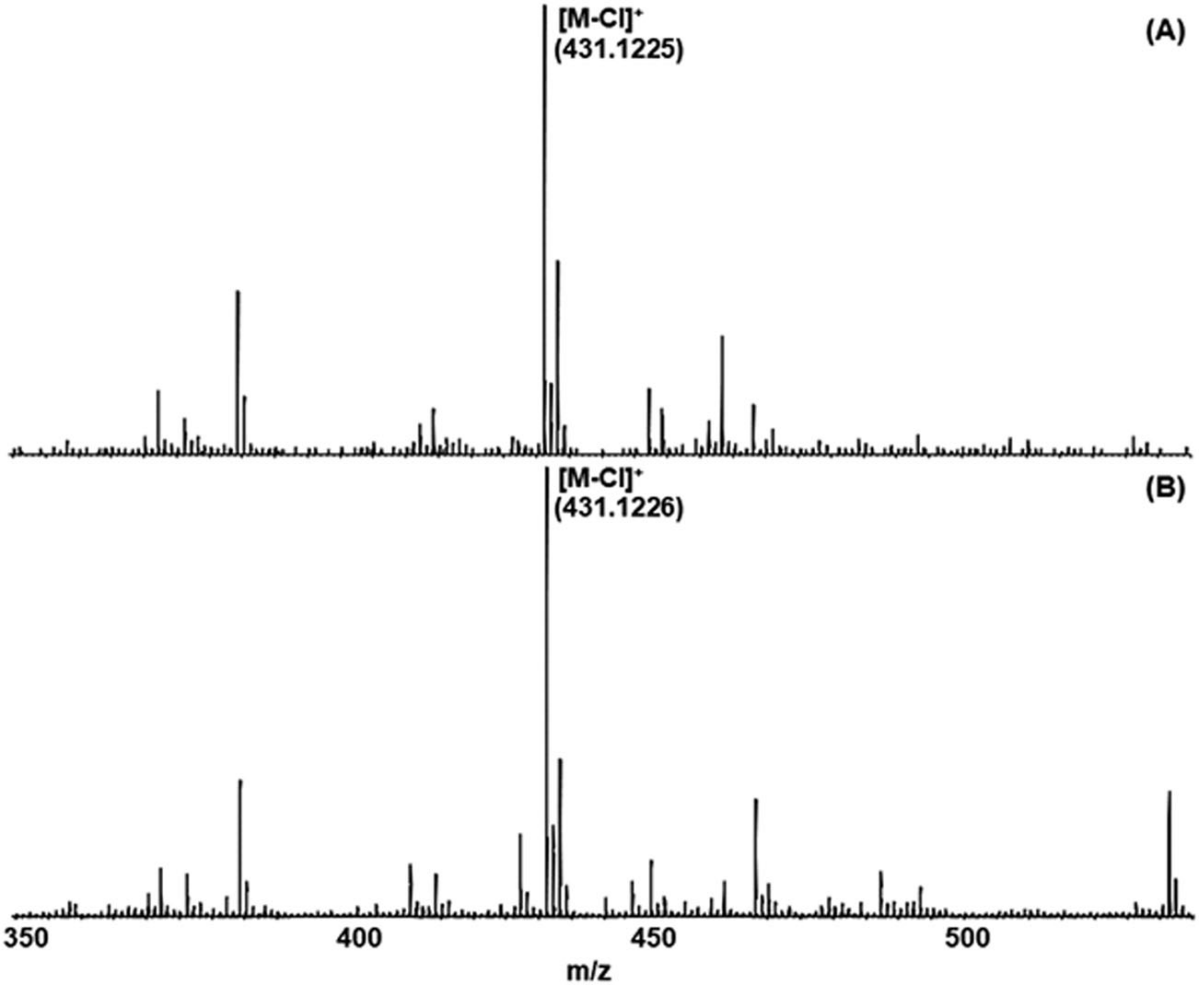
HR‐MALDI QTOF mass spectra of the dechlorinated molecule [M‐Cl]^+^ of **4** using A, DCTB (*trans*‐2‐[3‐(4‐*tert*‐butylphenyl)‐2‐methyl‐2‐propenylidene]malononitrile) and B, TTP (2,2′:5′,2″‐terthiophene) as matrices

Previous publications from Soltwisch et al reveal that high ion yields in MALDI‐MS can be obtained by the proper choice of the MALDI matrix matching the laser wavelength.^20^ Due to the high molar absorption of the Brønsted (Lewis) base matrices such as *trans*‐2‐[3‐(4‐*tert*‐butylphenyl)‐2‐methyl‐2‐propenylidene]malononitrile (DCTB) (*ɛ*_M_ = 31.410 M^−1^/cm) and TTP (ɛ_M_ = 33.500 M^−1^/cm) applying the laser wavelength of the Nd:YAG (355 nm) laser the instruments are equipped with were used.^8^, ^21^, ^22^ To figure out any differences concerning the performance of the matrices DCTB and TTP, measurements with both matrices were performed with all investigated samples. Figure 3 shows the mass spectra of the detected molecular ion species of compound **4** [Mn(PNP^NH^‐*i*Pr)Cl_2_] using DCTB (3A) and TTP (3B) as matrices for HR‐MALDI QTOF‐MS.

The comparative results of the use of the two different matrices demonstrated in Figure 3 show that there is no significant difference in the performance of these two matrices except for a few background peaks particularly in the higher mass range. They differ neither by different signal‐to‐noise ratios of the detected peak related to this analyte nor by ionization—identical molecular ion species can be detected in both spectra. This example is representative of all other compounds investigated.

### 3.2 Impact of the solvent choice

**FIGURE 4:**
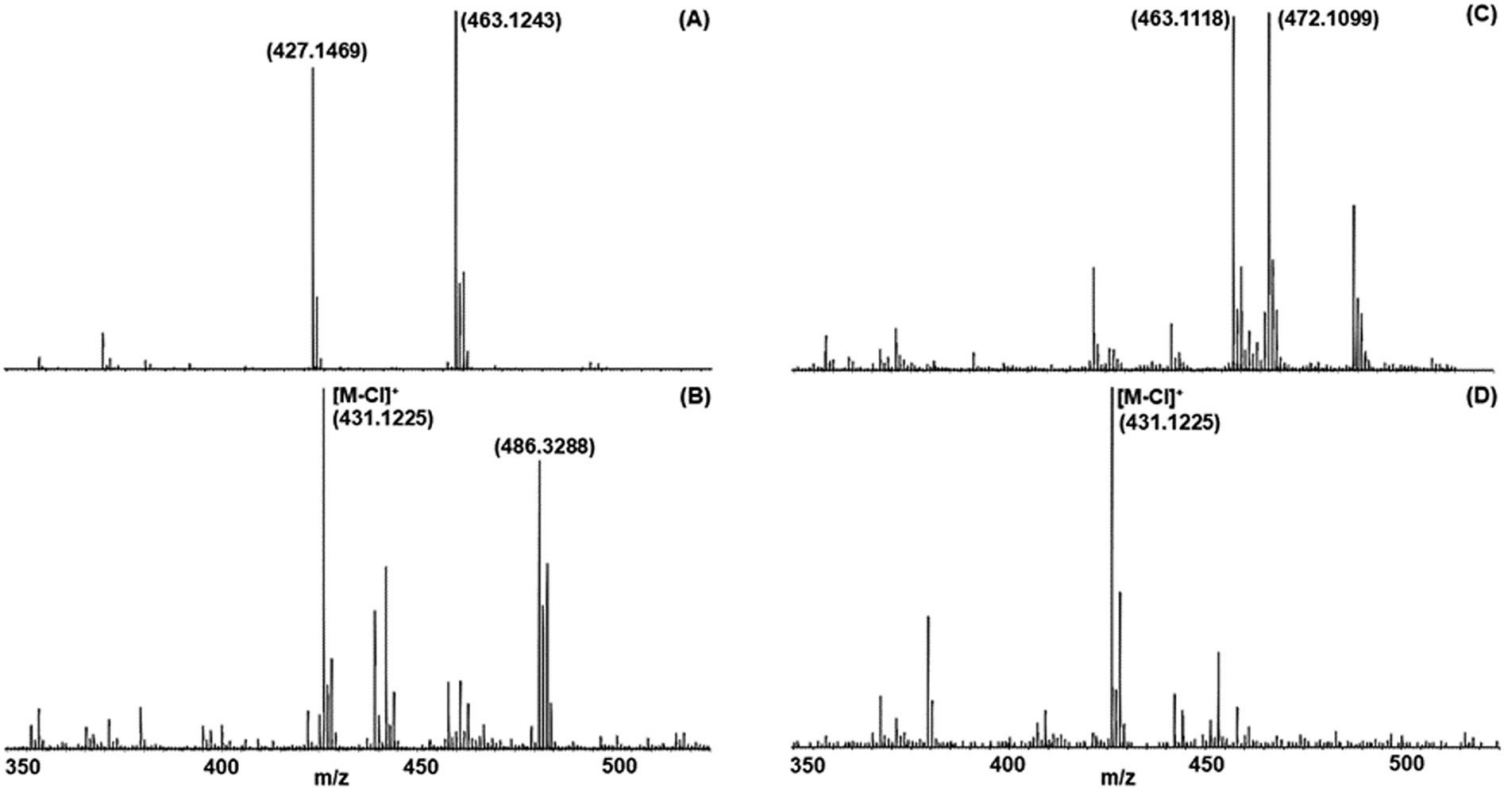
Impact of different solvent mixtures on the detected dechlorinated molecule [M‐Cl]^+^ of **4** by HR‐MALDI QTOF‐MS: A, acetonitrile/dichloromethane; B, methanol/chloroform; C, methanol/dichloromethane; and D, chloroform/1,2‐dichloropropane

Another aspect we are focusing on in this work is the influence of the solvents used for diluting the sample in the MALDI matrix solution for sample preparation. In contrast to sample preparation using ESI‐MS, where the choice of the solvent is strongly limited to polar solvents exhibiting a certain dipole moment as well as volatility, sample preparation for MALDI‐MS provides much more possibilities. If necessary, nonpolar solvents may also be used on the precondition that the matrix is also soluble in this solvent. Here, we compare the mass spectra of **4** [Mn(PNP^NH^‐*i*Pr)Cl_2_] (see Figure 4), with the four different solvent mixtures described in Table 2 (“Experimental” section). We have deliberately chosen those different solvent mixtures to demonstrate this phenomenon using a manganese complex as an example, as these are not easily soluble. Although the isotope pattern of the complex with the dechlorinated molecule [M‐Cl]^+^ can be identified with the solvent combinations methanol/chloroform (MeOH/TCM) (4B) and chloroform/1,2‐dichloropropane (TCM/DCP) (4D), no signal associated with this structure can be found in the spectrum of solvents ACN/DCM (4A) and MeOH/DCM (4C).

It is crucial to choose the proper solvent mixture individually for each compound to perform a successful analysis of organometallic compounds using MALDI‐MS.

Not only is the solubility of the complex important for the selection of the proper solvent combination, as this compound class is very sensitive, it is decisive that the sample does not decompose in the solution or that a ligand exchange with the solvent/solvent additive occurs. The ideal solvent mixture for the individual complexes is available in the supporting information (Table S1).

### 3.3 Intermediate‐pressure MALDI QTOF‐MS, high‐vacuum MALDI RTOF‐MS, and ESI QTOF‐MS—a comparison

**FIGURE 5:**
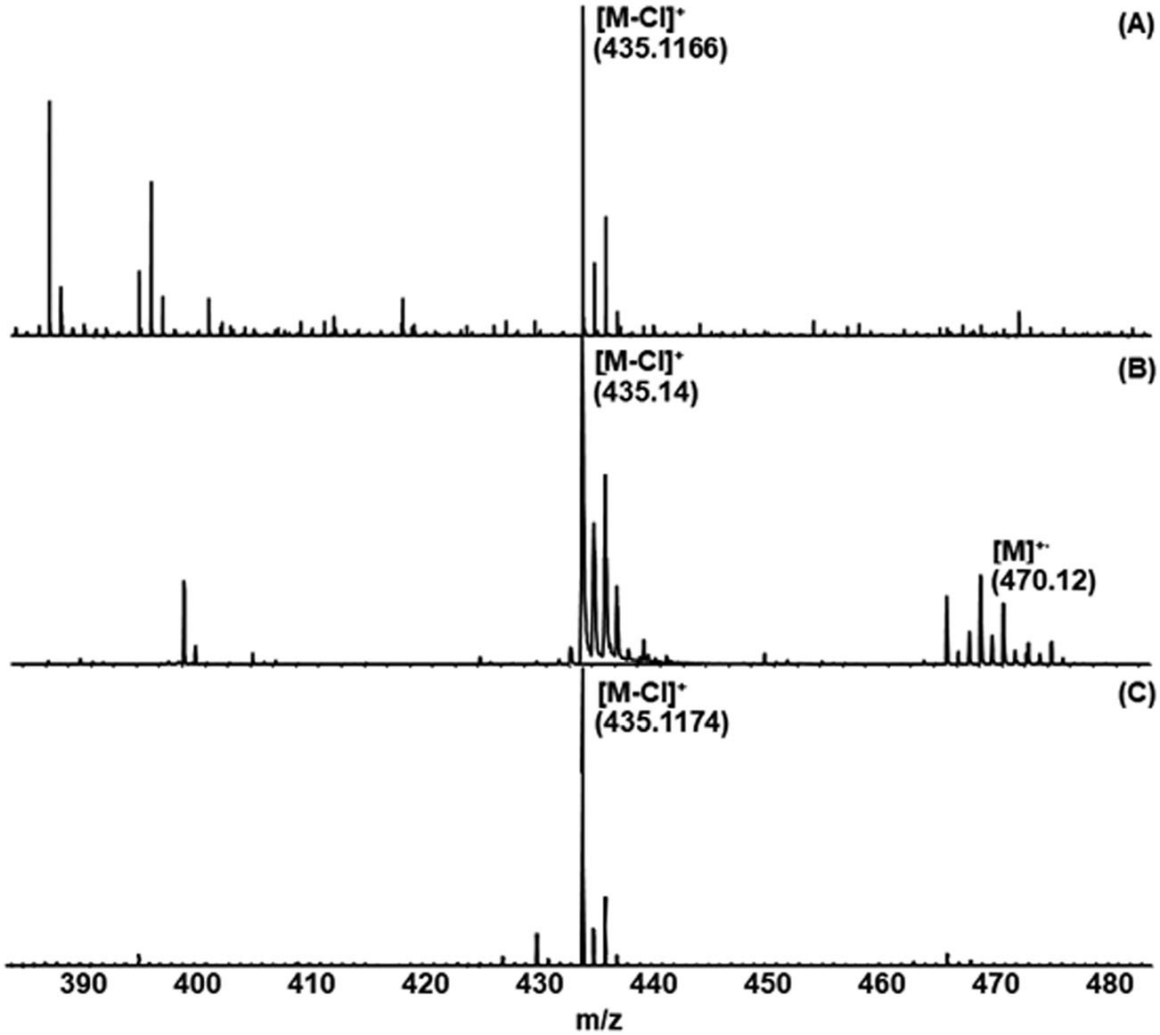
Comparison of the mass spectra of the dechlorinated molecule [M‐Cl]^+^ of **10** obtained with three instruments: A, HR‐ESI QTOF‐MS; B, vacuum‐MALDI TOF‐MS; and C, HR‐MALDI QTOF‐MS

To provide a direct comparison between the performance of different MALDI mass spectrometric techniques and the already‐established technique ESI‐MS for the analysis of “organometallic complexes,” the corresponding mass spectra of the investigated samples were directly compared with each other. A closer examination reveals that the ion related to the molecular ion species of **10** ([Co(PNP^NH^‐*i*Pr)Cl_2_]) is present in the mass spectrum as dechlorinated molecule [M‐Cl]^+^ ion with all instruments used in this study (see Figure 5). It is observed in the measurement using the ultrafleXtreme instrument that the [M]^+·^ peak could be detected in contrast to data obtained by either MALDI QTOF‐MS or ESI QTOF‐MS. This may be caused due to the fact that the ultrafleXtreme mass spectrometer is a reflectron TOF instrument, whereas the other instruments exhibit a coupling of quadrupole and reflectron TOF. This suggests the presumption that the [M]^+·^ ions exhibit metastable decomposition during the long flight path through the quadrupole section and via the orthogonal acceleration through the reflectron TOF section and no longer reach the detector.

**FIGURE 6:**
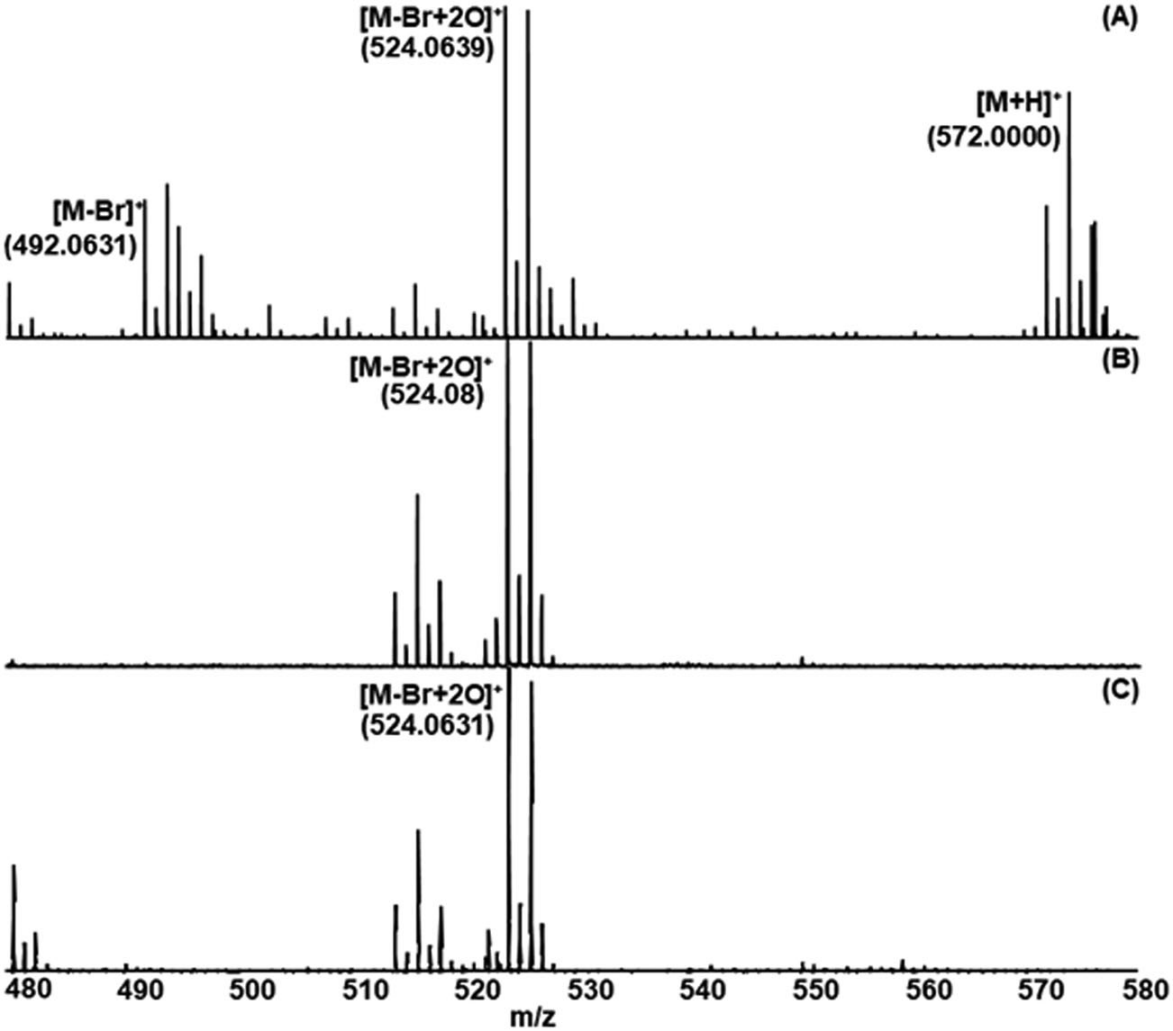
Comparison of the mass spectra of the molecular species of **7** obtained with three instruments: A, HR‐ESI QTOF‐MS; B, vacuum‐MALDI TOF‐MS; and C, HR‐MALDI QTOF‐MS

Investigating coordination compounds, rather unusual molecular species can also occur as explained in detail using **7** [Fe(Triaz^Me^‐*i*Pr)Br_2_] as an example. A closer examination of Figure 6 reveals that the complex could be identified as [M‐Br + 2O]^+^ in the spectra obtained by MALDI‐MS and ESI‐MS. In addition, ESI‐MS yields an [M + H]^+^ ion of significant abundance. Now, of course, the question may arise regarding how it is possible to detect an oxidized species like the one mentioned earlier. Most likely, this compound tends to add molecular oxygen (more likely forming a bis‐phosphine oxide than an iron–oxygen bonding). A possible explanation of this oxidation is that it might occur during sample handling/crystallization at atmospheric conditions until the drying process is completed on such a high‐surface area microcrystalline matrix structure. On the contrary, the occurrence of the [M‐Br + 2O]^+^ species in ESI‐MS could be explained by the interaction with atmospheric oxygen during the ESI process at elevated temperatures, whereas during MALDI sample preparation, an extended time window is tentatively responsible for the oxidation process. Similar observations for such structures were also observed after extended infusion times in an electrospray ion source (E. Pittenauer, unpublished observations). For all other compounds investigated in this study, observed ions related to the intact molecular ion (e.g., [M‐Cl]^+^, [M]^+·^, and [M + Na]^+^), see supporting information (Table S2).

## 4 CONCLUSION

This study reported on the successful identification of various first‐row transition metal (pincer) complexes using MALDI TOF‐MS. Examination of the analytes with different MALDI matrices, TTP and DCTB, showed no significant differences in the mass spectra observed. Investigating the influence of different solvent mixtures proved that the selection of the correct solvent mixture is crucial for a successful measurement. In this case various aspects have to be considered: the analyte should dissolve in the selected solvent without decomposing. In this respect, the sample preparation allows a much wider selection of different solvents than with ESI‐MS. This could provide an advantage in the analysis of samples, which are insoluble in purely polar solvents as methanol or acetonitrile. It should be further observed that MALDI‐MS is particularly suitable for neutral organometallic complexes, especially for those that can be dissolved only in nonpolar solvents. Moreover, the fact that the sample is in the solid state embedded in a matrix material can prove quite advantageous. ESI‐MS requires the sample to be in solution, which can be difficult especially for coordination compounds that decompose rapidly.

Despite its infrequent use, MALDI‐MS is an underestimated but powerful mass spectrometric technique that can be utilized as a complement or even as an alternative to ESI‐MS for the analysis of organometallic compounds.

## Supporting information




**FIGURE S1** Example for the nomenclature of a pincer complex
**FIGURE S2** Chemical structures of the matrices *trans*‐2‐[3‐(4‐*tert*‐butylphenyl)‐2‐methyl‐2‐propenylidene]malononitrile (DCTB) and 2,2′:5′,2″‐terthiophene (TTP)
**TABLE S1** Ideal solvent mixtures of investigated coordination compounds
**TABLE S2** Detected molecular ion species of investigated coordination compounds with different ionization techniques

## Data Availability

The data that support the findings of this study are available from the corresponding author upon reasonable request.

## References

[1] Crabtree RH . An organometallic future in green and energy chemistry? Organometallics. 2011;30(1):17‐19. doi:10.1021/om1009439

[2] Herrmann WA , Cornils B . Organometallic homogeneous catalysis—Quo vadis? Angew Chem Int Ed Engl. 1997;36(10):1048‐1067. doi:10.1002/anie.199710481

[3] Jirásko R , Holčapek M . Structural analysis of organometallic compounds with soft ionization mass spectrometry. Mass Spectrom Rev. 2011;30(6):1013‐1036. doi:10.1002/mas.20309 21104914

[4] Dyson PJ , McIndoe JS . Analysis of organometallic compounds using ion trap mass spectrometry. Inorg Chim Acta. 2003;354:68‐74. doi:10.1016/S0020-1693(03)00369-4

[5] Traeger JC . Electrospray mass spectrometry of organometallic compounds. Int J Mass Spectrom. 2000;200(1‐3):387‐401. doi:10.1016/S1387-3806(00)00346-8

[6] Vikse KL , Ahmadi Z , Scott McIndoe J . The application of electrospray ionization mass spectrometry to homogeneous catalysis. Coord Chem Rev. 2014;279:96‐114. doi:10.1016/j.ccr.2014.06.012

[7] Yunker LPE , Stoddard RL , McIndoe JS . Practical approaches to the ESI‐MS analysis of catalytic reactions. J Mass Spectrom. 2014;49(1):1‐8. doi:10.1002/jms.3303 24446256

[8] Bailey GA , Fogg DE . Confronting neutrality: Maximizing success in the analysis of transition‐metal catalysts by MALDI mass spectrometry. ACS Catal. 2016;6(8):4962‐4971. doi:10.1021/acscatal.6b01105

[9] Welker M . Proteomics for routine identification of microorganisms. Proteomics. 2011;11(15):3143‐3153. doi:10.1002/pmic.201100049 21726051

[10] Fuchs B , Schiller J . Application of MALDI‐TOF mass spectrometry in lipidomics. Eur J Lipid Sci Technol. 2009;111(1):83‐98. doi:10.1002/ejlt.200800223

[11] Tanaka K , Waki H , Ido Y , et al. Protein and polymer analyses up to m/z 100 000 by laser ionization time‐of‐flight mass spectrometry. Rapid Commun Mass Spectrom. 1988;2(8):151‐153. doi:10.1002/rcm.1290020802

[12] Wyatt MF . MALDI‐TOFMS analysis of coordination and organometallic complexes: A nic(h)e area to work in. J Mass Spectrom. 2011;46(7):712‐719. doi:10.1002/jms.1957 21744419

[13] Petroselli G , Mandal MK , Chen LC , et al. Mass spectrometry of rhenium complexes: A comparative study by using LDI‐MS, MALDI‐MS, PESI‐MS and ESI‐MS. J Mass Spectrom. 2012;47(3):313‐321. doi:10.1002/jms.2965 22431457

[14] Wyatt MF , Havard S , Stein BK , Brenton AG . Analysis of transition‐metal acetylacetonate complexes by matrix‐assisted laser desorption/ionization time‐of‐flight mass spectrometry. Rapid Commun Mass Spectrom. 2008;22:11‐18. doi:10.1002/rcm.3327 18050194

[15] Mastalir M , Glatz M , Stöger B , et al. Synthesis, characterization and reactivity of vanadium, chromium, and manganese PNP pincer complexes. Inorg Chim Acta. 2017;455:707‐714. doi:10.1016/j.ica.2016.02.064

[16] Mastalir M , Kirchner K . A triazine‐based Ni (II) PNP pincer complex as catalyst for Kumada–Corriu and Negishi cross‐coupling reactions. Monatshefte Für Chemie‐Chemical Mon. 2017;148(1):105‐109. doi:10.1007/s00706-016-1878-4 PMC5225227 28127096

[17] Mastalir M , Stöger B , Pittenauer E , Puchberger M , Allmaier G , Kirchner K . Air stable iron (II) PNP pincer complexes as efficient catalysts for the selective alkylation of amines with alcohols. Adv Synth Catal. 2016;358(23):3824‐3831. doi:10.1002/adsc.201600689

[18] Sládková K , Houška J , Havel J . Laser desorption ionization of red phosphorus clusters and their use for mass calibration in time‐of‐flight mass spectrometry. Rapid Commun Mass Spectrom. 2009;23(19):3114‐3118. doi:10.1002/rcm.4230 19714708

[19] Kussmann M , Nordhoff E , Rahbek‐Nielsen H , et al. Matrix‐assisted laser desorption/ionization mass spectrometry sample preparation techniques designed for various peptide and protein analytes. J Mass Spectrom. 1997;32(6):593‐601. doi:10.1002/(SICI)1096-9888(199706)32:6<593::AID-JMS511>3.0.CO;2-D

[20] Soltwisch J , Jaskolla TW , Hillenkamp F , Karas M , Dreisewerd K . Ion yields in UV‐MALDI mass spectrometry as a function of excitation laser wavelength and optical and Physico‐chemical properties of classical and halogen‐substituted MALDI matrixes. Anal Chem. 2012;84(15):6567‐6576. doi:10.1021/ac3008434 22803742

[21] Ingram AJ , Boeser CL , Zare RN . Going beyond electrospray: Mass spectrometric studies of chemical reactions in and on liquids. Chem Sci. 2016;7(1):39‐55. doi:10.1039/C5SC02740C 28757996 PMC5508663

[22] McCarley TD , McCarley RL , Limbach PA . Electron‐transfer ionization in matrix‐assisted laser desorption/ionization mass spectrometry. Anal Chem. 1998;70(20):4376‐4379. doi:10.1021/ac980527i

