# Supplementary material for: Characterization of selected organometallic compounds by electrospray ionization‐ and matrix‐assisted laser desorption/ionization‐mass spectrometry using different types of instruments: Possibilities and limitations
Source: Rapid Commun Mass Spectrom. 2022 Mar 21;36(10):e9281. doi: 10.1002/rcm.9281 (PMC9286352; doi:10.1002/rcm.9281)
Supplement: Supplementary file 1 — FIGURE S1 Example for the nomenclature of a pincer complex FIGURE S2 Chemical structures of the matrices trans‐2‐[3‐(4‐tert‐butylphenyl)‐2‐methyl‐2‐propenylidene]malononitrile (DCTB) and 2,2′:5′,2″‐terthiophene (TTP) TABLE S1 Ideal solvent mixtures of investigated coordination compounds TABLE S2 Detected molecular ion species of investigated coordination compounds with different ionization techniques [file RCM-36-0-s001.docx]

**Supporting Information**

**CHARACTERIZATION OF SELECTED ORGANOMETALLIC COMPOUNDS BY ESI- AND MALDI-MS USING DIFFERENT TYPES OF INSTRUMENTS: POSSIBILITIES AND LIMITATIONS**

Sarah Fleissner^1^, Ernst Pittenauer^2^, Jan Pecak^1^, Karl Kirchner^1^

^1^Institute of Applied Synthetic Chemistry, Vienna University of Technology, Vienna; Austria

^2^Institute of Chemical Technologies and Analytics, Vienna University of Technology, Vienna; Austria

**S1 Ligand nomenclature**

**Figure S1.** Example for the nomenclature of a pincer complex

**S2 Matrix structures**

**Figure S2**. Chemical structures of the matrices trans-2-[3-(4-tert-butylphenyl)-2-methyl-2-propenylidene] malononitrile (DCTB) and 2,2':5',2"-terthiophene (TTP)

**S3 Solvent Mixtures**

**Table S1.** Ideal solvent mixtures of investigated coordination compounds.

| No. | Solvent Mixtures |
| --- | --- |
| 1 | MeOH/DCM |
| 2 | ACN/DCM |
| 3 | MeOH/DCM |
| 4 | TCM/DCP |
| 5 | TCM/DCP |
| 6 | MeOH/DCM |
| 7 | MeOH/TCM |
| 8 | TCM/DCP |
| 9 | TCM/DCP |
| 10 | MeOH/DCM |
| 11 | MeOH/DCM |
| 12 | MeOH/DCM |

**S4 Detected Molecular Ion Species**

**Table S2.** Detected molecular ion species of investigated coordination compounds with different ionization techniques.

| No. | Detected molecular ion species | | |
| --- | --- | --- | --- |
|  | **Synapt G2 HDMS** | **ultrafleXtreme** | **Agilent 6545 QTOF** |
| 1 | [M-Cl]^+^ | [M-Cl]^+^ | (-) |
| 2 | [M-Cl]^+^ | [M-Cl]^+^ | (-) |
| 3 | [M-3CO+Cl]^+^ | [M-3CO+Cl]^+^ | [M]^+·^ |
| 4 | [M-Cl]^+^ | [M-Cl]^+^ | (-) |
| 5 | [M-Br]^+^ | [M-Br]^+^ | (-) |
| 6 | [M+Na]^+^ | [M+Na]^+^ | [M+Na]^+^ |
| 7 | [M-Br+2O]^+^ | [M-Br+2O]^+^ | [M-Br+2O]^+^, [M+H]^+^ |
| 8 | [M-Br+2O]^+^ | [M-Br+2O]^+^ | [M-Br+2O]^+^, [M+H]^+^ |
| 9 | [M]^+·^ | [M]^+·^ | [M-Cl]^+^ |
| 10 | [M-Cl]^+^ | [M-Cl]^+^, [M]^+·^ | [M-Cl]^+^ |
| 11 | [M-Cl]^+^ | [M-Cl]^+^, [M]^+·^ | [M-Cl]^+^ |
| 12 | [M-Cl]^+^ | [M-Cl]^+^, [M]^+·^ | [M-Cl]^+^ |
